# A novel intraperitoneal therapy for gastric cancer with DFP‐10825, a unique RNAi therapeutic targeting thymidylate synthase, in a peritoneally disseminated xenograft model

**DOI:** 10.1002/cam4.2598

**Published:** 2019-10-14

**Authors:** Hidenori Ando, Masakazu Fukushima, Kiyoshi Eshima, Taichi Hasui, Taro Shimizu, Yu Ishima, Cheng‐Long Huang, Hiromi Wada, Tatsuhiro Ishida

**Affiliations:** ^1^ Department of Pharmacokinetics and Biopharmaceutics Institute of Biomedical Sciences Tokushima University Tokushima Japan; ^2^ Department of Cancer Metabolism and Therapy Institute of Biomedical Sciences Tokushima University Tokushima Japan; ^3^ Delta‐Fly Pharma Inc Tokushima Japan; ^4^ Department of Thoracic Surgery Faculty of Medicine Kyoto University Kyoto Japan

**Keywords:** DFP‐10825, gastric cancer, peritoneal dissemination, RNAi therapeutic, S‐1, thymidylate synthase (TS)

## Abstract

**Purpose:**

In advanced gastric cancer, peritoneal dissemination is a life‐threatening mode of metastasis. Since the treatment options with conventional chemotherapy remain limited, any novel therapeutic strategy that could control such metastasis would improve the outcome of treatment. We recently developed a unique RNA interference therapeutic regimen (DFP‐10825) consisting of short hairpin RNA against thymidylate synthase (TS shRNA) and cationic liposomes. The treatment with DFP‐10825 has shown remarkable antitumor activity in peritoneally disseminated human ovarian cancer–bearing mice via intraperitoneal administration. In this study, we expanded DFP‐10825 to the treatment of peritoneally disseminated gastric cancer.

**Methods:**

DFP‐10825 was administered intraperitoneally into mice with intraperitoneally implanted human gastric cancer cells (MKN45 or NCI‐N87). Antitumor activity and host survival benefits were monitored. Intraperitoneal distribution of fluorescence‐labeled DFP‐10825 was monitored in this MKN45 peritoneally disseminated mouse model.

**Results:**

Intraperitoneal injection of DFP‐10825 suppressed tumor growth in two peritoneally disseminated cancer models (MKN45 and NCI‐N87) and increased the survival time of the MKN45 model without severe side effects. Throughout the treatment regimen, no significant body weight loss was associated with the administration of DFP‐10825. Interestingly, after intraperitoneal injection, fluorescence‐labeled DFP‐10825 retained for more than 72 hours in the peritoneal cavity and selectively accumulated in disseminated tumors.

**Conclusions:**

Intraperitoneal injection of DFP‐10825 demonstrated effective antitumor activity without systemic severe adverse effects via the selective delivery of RNAi molecules into disseminated tumors in the peritoneal cavity. Our current study indicates that DFP‐10825 could become an alternative option to improve the outcomes of patients with peritoneally disseminated gastric cancer.

## Graphical abstract

Intraperitoneal DFP‐10825 showed effective antitumor activity in peritoneally disseminated human gastric cancer–bearing mice. As an alternative treatment regimen, DFP‐10825 has the potential to improve the outcomes of patients with peritoneally disseminated gastric cancer.

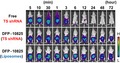

## 1 INTRODUCTION

Gastric cancer is the fifth most common cancer around the world and is the third leading cause of cancer‐related death.^1^ In advanced gastric cancers, peritoneal dissemination is the most life‐threatening development, because it frequently causes severe clinical symptoms such as malignant ascites and intestinal obstruction.^2^, ^3^, ^4^ For the treatment of peritoneally disseminated gastric cancer, cytoreductive surgery and systemic chemotherapy are the standard option in clinical cases. Several reports have indicated, however, that systemic chemotherapy is ineffective for peritoneal dissemination, because the systemic anticancer agents cannot reach into the lesion in the peritoneal cavity where the disseminated tumors develop.^5^ To conquer this problem, intraperitoneal chemotherapy has recently been introduced as a novel strategy to treat peritoneally disseminated gastric cancer.^6^, ^7^ Intraperitoneal chemotherapy is expected to directly expose disseminated tumors to treatment drugs, which would simplify the process of controlling drug concentration in the peritoneal cavity. In fact, several clinical studies have elucidated the advantages of intraperitoneal chemotherapy for gastric peritoneal dissemination.^8^, ^9^ Nevertheless, no effective treatment has yet improved the survival rates of patients, because intraperitoneal anticancer agents tend to be rapidly removed from the peritoneal cavity via the circulatory system, which prevents the administration of a therapeutic concentration to the peritoneal cavity within a sufficient therapeutic window.^10^ Accordingly, a novel therapeutic approach that would control peritoneal dissemination in advanced gastric cancer is required to increase the survival rates of patients.

We recently developed the DFP‐10825 formulation, which is a unique RNAi molecule consisting of shRNA against thymidylate synthase (TS) and a cationic liposome, which is intended for local administration^11^ and systemic injection.^12^ Thymidylate synthase is well‐known as an important enzyme that is involved in the DNA synthesis/repair of cancer cells and tumor malignancy^13^, ^14^, ^15^; fluoropyrimidines are designed to primarily target TS,^16^ along with its derivatives.^17^ Systemic injection of DFP‐10825 improved the therapeutic efficacy of S‐1 orally administered in a colorectal cancer xenograft mouse model.^18^ S‐1 is an oral anticancer drug combined of 1‐(2‐tetrahydrofuryl)‐5‐fluorouracil (tegafur), 5‐chloro‐2,4‐dihydropyrimidine, and potassium oxonate in a molar ratio of 1:0.4:1, which is standard care for gastric cancer. An injection of DFP‐10825 also improved the therapeutic effect of pemetrexed (PMX) intraperitoneally administered to a subcutaneous malignant mesothelioma xenograft mouse model.^12^ In addition, intrapleural injection of DFP‐10825 increased the therapeutic effect of PMX peritoneally administered to a malignant pleural mesothelioma orthotropic xenograft mouse model via suppression of TS expression in implanted mesothelioma cells.^11^

To expand the therapeutic application of DFP‐10825, in this study, we demonstrated the antitumor activity of intraperitoneally delivered DFP‐10825 on a peritoneally disseminated gastric cancer mouse model. The model mice were treated with either DFP‐10825 or S‐1. As we expected, the treatment with oral S‐1 did not produce the antitumor effects on peritoneally disseminated tumors. In contrast, the intraperitoneal treatment with DFP‐10825 showed superior therapeutic effects without any systemic adverse events. The present study shows that intraperitoneal delivery of DFP‐10825 could be a feasible therapeutic approach to treat peritoneally disseminated gastric cancer.

## 2 MATERIALS AND METHODS

### 2.1 Materials

Thymidylate synthase shRNA for clinical use produced by a scaled‐up rate of synthesis was obtained from Nitto‐Denko Avecia (Milford). The TS shRNA sequence was 5′‐GUA ACA CCA UCG AUC AUG AUA GUG CUC CUG GUU GUC AUG AUC GAU GGU GUU ACU U‐3′. Fluorescently labeled Alexa750 TS shRNA was purchased from GeneDesign. Dioleoyl phosphatidylethanolamine (DOPE) and dioleoyl phosphatidylcholine (DOPC) were kindly provided by NOF. A cationic lipid, O,O′‐ditetradecanoyl‐*N*‐(α‐trimethyl ammonio acetyl) diethanolamine chloride (DC‐6‐14), was purchased from Sogo Pharmaceutical. The anticancer agent S‐1 was obtained from Taiho Pharmaceutical. 1,1′‐Dioctadecyl‐3,3,3′,3′‐tetramethylindotricarbocyanine iodide (DiR) and RNase‐free water (UltraPure^TM^ DNase/RNase‐Free Distilled Water) were purchased from Life Technologies. D‐luciferin potassium salt was purchased from FUJIFILM Wako Pure Chemical Corporation. All other reagents were of analytical grade.

### 2.2 Preparation of TS shRNA‐lipoplex (DFP‐10825)

Cationic liposome composed of DOPE:DOPC:DC‐6‐14 (3:2:5 molar ratio) was prepared using a previously described method.^11^ Thymidylate synthase shRNA and cationic liposome were mixed at a molar ratio of 1:2000 (shRNA:lipid), and the mixture was vigorously vortexed for 10 minutes at room temperature to form a TS shRNA/cationic liposome complex (TS shRNA‐lipoplex, DFP‐10825).

### 2.3 Preparation of a peritoneally disseminated gastric cancer mouse model

MKN45 human gastric carcinoma was purchased from the RIKEN BioResource Center (RCB1001). NCI‐N87 human gastric carcinoma expressing firefly luciferase was purchased from Summit Pharmaceuticals International. The cells were cultured in RPMI‐1640 medium (FUJIFILM Wako Pure Chemical Corporation) supplemented with 10% of heat‐inactivated fetal bovine serum (Corning), 100 units/mL penicillin, and 100 μg/mL streptomycin (ICN Biomedicals) in a 5% CO_2_/air incubator at 37°C.

BALB/c *nu/nu* mice (male, 5 weeks old) were purchased from Japan SLC. The experimental animals were allowed free access to water and mouse chow and were housed under controlled environmental conditions (constant temperature and humidity, and a 12‐hour dark‐light cycle). All animal experiments were evaluated and approved by the Animal and Ethics Review Committee of Tokushima University. To prepare the peritoneal dissemination mouse model, either MKN45 cells or NCI‐N87 cells were intraperitoneally injected into BALB/c *nu/nu* mice (5 × 10^6^ cells/mouse). The development of a MKN45 peritoneally disseminated mouse model was confirmed by a >1.5 g decrease in body weight from Day 0 to Day 7 post‐tumor implantation. Development of the NCI‐N87 peritoneally disseminated mouse model was confirmed using an in vivo imaging system (IVIS; Xenogen) by intraperitoneal injection with 100 μL of 7.5 mg/mL D‐luciferin potassium salt followed by anesthesia administered by isoflurane inhalation. At 5 minutes post‐injection, bioluminescence of the disseminated tumors was observed with a CCD camera (exposure time was fixed at 30 seconds). The region of interest in bioluminescence was calculated and displayed as a photon count (photons/s).

### 2.4 Survival and increased life span for a DFP‐10825‐treated MKN45 peritoneally disseminated mouse model

MKN45 peritoneally disseminated model mice were intraperitoneally injected with five injections of DFP‐10825 (2.5, 5, 10, or 20 µg TS shRNA/mouse/d) twice weekly from Day 7 post‐inoculation of tumor cells. The antitumor effect was assessed in terms of both the mean survival time (MST [day]) and the increased life span (ILS [%]). The MST was identified with recording the mortality on a daily basis, and the ILS for the treatment groups was calculated using the following formula:

$$\text{ILS}=[\left(\text{MST}-{\text{MST}}_{\text{control}}/{\text{MST}}_{\text{control}}\right)]\times 100.$$

### 2.5 Tumor growth inhibitory effect of DFP‐10825 in a peritoneally disseminated tumor model

Luciferase‐expressing peritoneally disseminated mouse models were prepared by intraperitoneal injection with either luciferase‐expressing MKN45 cells or luciferase‐expressing NCI‐N87 cells. Peritoneally disseminated model mice were intraperitoneally injected with either DFP‐10825 (20 µg/mouse/d, four or five injections once every 3 days, i.p.) or S‐1 (3.5 mg tegafur/kg/d, 14 doses every day, p.o.) from Day 7 post‐tumor implantation. The dose and the treatment schedule of S‐1 was adopted by reference to the paper from the other group.^19^ At selected time points, luciferase activity of the disseminated tumors was monitored with IVIS. In vivo imaging was performed as described above. The antitumor effect was assessed in terms of both the bioluminescent intensity (BLI) and the tumor growth inhibition (TGI [%]). The BLI was estimated based on the luciferase activity of peritoneally disseminated tumors, as determined by IVIS. The TGI was calculated using the following formula:

$$\text{TGI}=\left(1{-}^{\text{BLI}}{/}_{{\text{BLI}}_{\text{control}}}\right)\times 100.$$

### 2.6 Biodistribution of intraperitoneally injected DFP‐10825 in a peritoneally disseminated mouse model

To follow the biodistribution of TS shRNA and cationic liposomes, the DFP‐10825 formulation was labeled with either Alexa750‐labeled TS shRNA or hydrophobic DiR (a lipid membrane marker). Fluorescently labeled DFP‐10825 (20 µg TS shRNA/mouse) was intraperitoneally injected into a MKN45 peritoneally disseminated mouse model. At selected time points post‐injection (5, 10, and 30 minutes; 1, 3, 6, 12, 24, 48, and 72 hours), the distribution of DFP‐10825 was visualized using IVIS. At a final time point, the mice were euthanized, and the tumor and organs including heart, lung, liver, spleen, and kidney were collected. Then, the accumulation of fluorescently labeled DFP‐10825 was observed using IVIS with a CCD camera (exposure time was fixed at 30 seconds) under the following filter setting: Ex 710 nm, Em 760 nm.

### 2.7 Statistical analysis

Statistical differences between the groups were evaluated by ANOVA with the Tukey post hoc test using the BellCurve for Excel software (Social Survey Research Information). All values are reported as the mean ± SD. The levels of significance were set at *P < .05, **P < .01 vs. control, ^#^ *P* < .05 vs S‐1 treatment, and ^$$^ *P* < .01 vs 2.5 µg TS shRNA.

## 3 RESULTS

### 3.1 Therapeutic effect of DFP‐10825 on the MKN45 peritoneally disseminated mouse model: dose dependency

**Figure 1:**
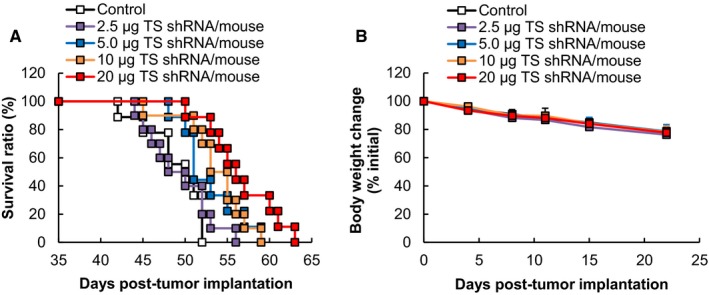
Therapeutic effect of DFP‐10825 on a MKN45 peritoneally disseminated mouse model. MKN45 peritoneally disseminated model mice were intraperitoneally injected with five injections of DFP‐10825 (2.5, 5, 10, or 20 µg thymidylate synthase [TS] short hairpin RNA [shRNA]/mouse/d) twice weekly from Day 7 post‐tumor implantation. A, Survival periods for the mice were monitored daily (n = 9‐10). B, Body weight changes of the mice were monitored twice weekly. The data are represented as the mean ± SD

To study the therapeutic effect of DFP‐10825 on peritoneally disseminated gastric cancer, different doses of DFP‐10825 (2.5, 5.0, 10, or 20 µg TS shRNA/mouse/d) were intraperitoneally injected into a MKN45 peritoneally disseminated mouse model twice weekly. The intraperitoneal injection of DFP‐10825 prolonged the survival times of the mice, compared with the untreated control mice (Figure 1A). The MST and ILS were increased in a dose‐dependent manner (Table 1). The body weight of the treated mice gradually decreased with each dose of DFP‐10825, and yet, it was comparable to that of the untreated control mice (Figure 1B). These results indicate that intraperitoneally injected DFP‐10825 exerts potent therapeutic efficacy on peritoneally disseminated gastric cancer without the severe adverse effects.

**Table 1:** MST and ILS of treated a MKN45 peritoneally disseminated mouse model from the data of Figure 1A

Treatment	Doses	MST (d)	ILS (%)
Control	—	48.9 ± 3.5	—
DFP‐10825	2.5 µg TS shRNA	49.3 ± 3.9	0.8
	5.0 µg TS shRNA	52.8 ± 3.6	8.0
	10 µg TS shRNA	53.6 ± 3.9	9.6
	20 µg TS shRNA	56.6 ± 4.2**^,$$^	15.7

Note

MST (d) and ILS (%) were determined from the data shown in Figure 1A. The MSTs are represented as the mean ± SD (n = 9‐10, **P < .01 vs control, ^$$^ *P* < .01 vs 2.5 µg TS shRNA). The ILSs were calculated using a formula described in Section 2.

Abbreviations: ILS, increased life span; MST, mean survival time; TS, thymidylate synthase.

### 3.2 DFP‐10825 suppression of tumor growth in the MKN45 peritoneally disseminated mouse model

**Figure 2:**
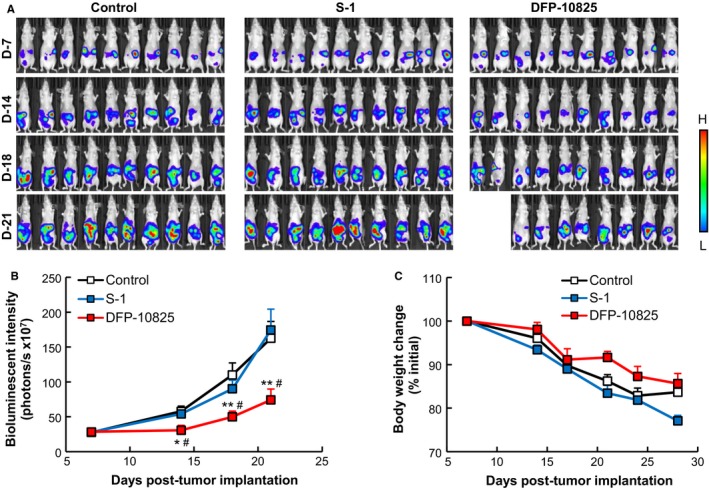
Tumor growth suppressive effect of DFP‐10825 in a MKN45 peritoneally disseminated mouse model. Luciferase‐expressing MKN45 peritoneally disseminated model mice were injected with either DFP‐10825 (20 µg/mouse/d, four doses once every 3 d, i.p.) or S‐1 (3.5 mg tegafur/kg/d, 14 doses once every day, p.o.) from Day 7 post‐tumor implantation. A, Bioluminescence of the disseminated tumors was monitored with in vivo imaging system at selected time points (Days 7, 14, 18, and 21 post‐tumor implantation). Two of 10 mice in the DFP‐10825‐treated group died accidently due to excessive anesthesia. B, Bioluminescent intensities of the disseminated tumors were calculated from the images in Figure 2A. The data are represented as the mean ± SD (n = 10, *P < .05, **P < .01 vs control, ^#^ *P* < .05 vs S‐1). C, Body weight changes of the mice were monitored at selected time points. The data are represented as the mean ± SD

The suppressive effect that DFP‐10825 exerted on tumor growth was compared with that of conventional standard chemotherapy for gastric cancer, S‐1. The growth of implanted tumor cells in the peritoneal cavity was monitored with IVIS by tracing the luciferase activity of the tumor cells (Figure 2A). Regardless of the treatment group, intraperitoneal luciferase activity regarding tumor growth was detected in all mice. In the untreated control group, the luciferase activity increased with increasing time following implantation (Figure 2A,B). In the S‐1‐treated mice, the luciferase activity also increased with an increase in time and the luciferase activity at each time point was almost the same as that for the control mice (Figure 2A,B). This observation indicates that S‐1 showed no tumor growth inhibition under our current experimental conditions. On the other hand, in DFP‐10825‐treated mice, the intraperitoneal luciferase activity increased slowly, and at each time point, with the exception of the earliest point, the luciferase activity was smaller than that for either the S‐1‐treated or the control mice (Figure 2A,B). Body weights of the mice treated with both S‐1 and DFP‐10825 gradually decreased, but were comparable to that of the untreated control mice (Figure 2C). These results indicate that intraperitoneally injected DFP‐10825 showed TGI without severe adverse effects, which is consistent with the finding shown in Figure 1.

### 3.3 Tumor growth suppressive effect of DFP‐10825 on a NCI‐N87‐LUC peritoneally disseminated mouse model

**Figure 3:**
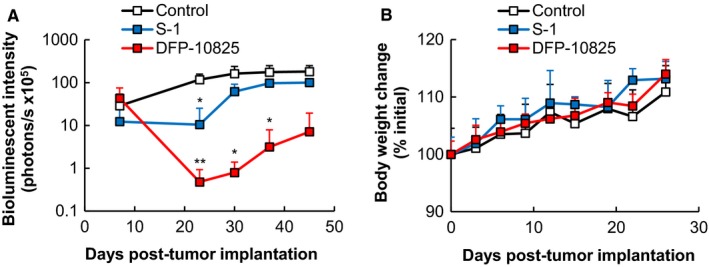
Tumor growth suppressive effect of DFP‐10825 in a NCI‐N87 peritoneally disseminated mouse model. Luciferase‐expressing NCI‐N87 peritoneally disseminated model mice were injected with either DFP‐10825 (20 µg/mouse/d, five injections once every 3 d, i.p.) or S‐1 (3.5 mg tegafur/kg/d, 14 doses once every day, p.o.) from Day 7 post‐tumor implantation. A, Bioluminescent intensities of the disseminated tumors were calculated from the data imaged using in vivo imaging system at selected time points (Days 7, 23, 30, 37, and 45 post‐tumor implantation). The data are represented as the mean ± SD (n = 2‐3, *P < .05, **P < .01 vs control). B, Body weight change of the mice was monitored at selected time points. The data are represented as the mean ± SD

To further confirm the tumor growth suppression effect of DFP‐10825, we next prepared a peritoneally disseminated gastric cancer model with another cell line, NCI‐N87 human gastric carcinoma. NCI‐N87 cells gradually develop in the peritoneum after intraperitoneal implantation and induce the accumulation of tumoral ascites fluid in the peritoneal cavity,^20^ which is highly consistent with the clinical conditions of peritoneal dissemination. Either DFP‐10825 (20 µg/mouse/d, five injections once every 3 days, i.p.) or S‐1 (3.5 mg tegafur/kg/d, 14 doses once every day, p.o.) was administered into the model mice. Peritoneal luciferase activity was detected in all mice when the treatments were begun (Figure 3A), indicating that the NCI‐N87 peritoneally disseminated tumors had begun to grow in the peritoneum. In the untreated control mice, peritoneal luciferase activity gradually increased with time. In the S‐1‐treated mice, the peritoneal luciferase activity was maintained at a constant level during the treatment period (Day 7‐Day 21) and then began to increase to a controlled level. The TGI value for the S‐1 treatment group was 44.7%. This indicates that S‐1 treatment suppressed the tumor growth in this gastric cancer disseminated model. In the DFP‐10825‐treated mice, the luciferase activity was somewhat decreased during the treatment period (Day 7‐Day 21) and then began to increase. For the DFP‐10825‐treated mice, however, the luciferase activity at Day 45 was much smaller than that of both the untreated mice and the S‐1‐treated mice. The TGI value for DFP‐10825 was 96%. All mice in both treatment groups showed a comparable body weight increase (Figure 3B). These results indicate that the intraperitoneal injection of DFP‐10825 showed a superior tumor growth suppressive effect without severe side effects in yet another gastric cancer peritoneally disseminated mouse model.

### 3.4 Biodistribution of intraperitoneally injected DFP‐10825 in a MKN45 peritoneally disseminated mouse model

**Figure 4:**
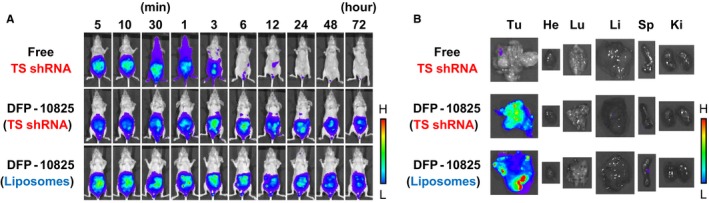
Biodistribution of intraperitoneally injected DFP‐10825 in a MKN45 peritoneally disseminated mouse model. MKN45 peritoneally disseminated model mice were intraperitoneally injected with free Alexa750‐labeled TS short hairpin RNA (shRNA), DFP‐10825 containing Alexa750‐labeled TS shRNA, or DFP‐10825 containing 1,1′‐dioctadecyl‐3,3,3′,3′‐tetramethylindotricarbocyanine iodide (DiR) (20 µg thymidylate synthase [TS] shRNA/mouse). A, The fluorescence of Alexa750‐labeled TS shRNA or DiR in the formulation was monitored with in vivo imaging system (IVIS) at selected time points (5, 10, and 30 min; 1, 3, 6, 12, 24, 48, and 72 h post‐injection). B, After preserving an image at 72 h post‐injection, tumors (Tu) and organs including heart (He), lung (Lu), liver (Li), spleen (Sp), and kidney (Ki) were harvested. The fluorescence of Alexa750‐labeled TS shRNA or DiR in each of the tissues was visualized with IVIS

To track the biodistribution of DFP‐10825 following intraperitoneal injection, two types of fluorescently labeled DFP‐10825 were prepared; one formulation contained Alexa750‐labeled TS shRNA and the other contained a hydrophobic DiR‐labeled liposomal membrane. These fluorescently labeled DFP‐10825 samples were intraperitoneally injected into different MKN45 peritoneally disseminated model mice, and the in vivo distributions were visualized using IVIS (Figure 4A). Free Alexa750‐labeled TS shRNA was retained within the peritoneal cavity at earlier time points, then distributed over the body, and rapidly eliminated from the body at 6 hours post‐injection. DFP‐10825 containing either Alexa750‐labeled TS shRNA or DiR was retained in the peritoneal cavity without distribution over the body at 72 hours post‐injection. At 72 hours post‐injection, the tumors and the normal organs, including heart, lung, liver, spleen, and kidney, were harvested, and the accumulation of DFP‐10825 in these tissues was observed (Figure 4B). Free Alexa750‐labeled TS shRNA showed no accumulation in any tissues. Interestingly, DFP‐10825 containing either Alexa750‐labeled TS shRNA or DiR was selectively accumulated in the peritoneally disseminated tumor masses without accumulation in any abdominal organs. These results indicate that intraperitoneally injected DFP‐10825 was retained in the peritoneal cavity for extended periods of time and was selectively accumulated in the peritoneally disseminated tumors, which efficiently delivered TS shRNA to the disseminated tumors and resulted in TS shRNA‐mediated RNAi.

## 4 DISCUSSION

Peritoneal dissemination is frequently detected in patients with advanced gastrointestinal cancer,^21^, ^22^ which causes a poor prognosis for the patient. A novel therapeutic approach to controlling peritoneal dissemination in advanced gastric cancer is urgently needed, because it would increase both the quality of life and the survival of patients. Thus far, however, neither systemic chemotherapy nor intraperitoneal chemotherapy has shown promising clinical benefits.^6^, ^23^, ^24^ In the current study, we showed that intraperitoneal injection of DFP‐10825 induced desired tumor growth suppression in two different types of peritoneally disseminated gastric cancer models (MKN45 and NCI‐N87) (Figures 2A,B and 3A) and increased the survival time of a MKN45 peritoneally disseminated gastric cancer model (Figure 1A) without severe side effects (Figures 1B, 2C and 3B). Our DFP‐10825 model could become an alternative approach for improving the outcome of patients with peritoneally disseminated gastric cancer.

Thymidylate synthase has been recognized as a rate‐limiting enzyme in de novo pyrimidine biosynthesis and plays a key role in the metabolism of folate and deoxythymidine for DNA synthesis and repair.^25^, ^26^ Several studies reported that the TS‐expression level is related to the therapeutic performance in clinical cases for the treatment of gastric cancer; the higher the TS expression, the poorer the therapeutic performance.^27^, ^28^ Hence, TS is considered a critical target for the treatment of malignantly advanced gastric cancer. The Japanese guidelines on gastric cancer treatment (updated in 2014)^29^ list S‐1 plus cisplatin (CDDP) as the first‐line chemotherapy for advanced gastric cancer. It is well‐known that TS is a target enzyme of 5‐FU derived from S‐1. DFP‐10825 is an RNAi therapeutic composed of TS shRNA conjugated with cationic liposomes that has downregulated TS in cancer xenograft models.^11^, ^30^ Accordingly, DFP‐10825 should be a feasible approach to control the malignancy of metastatic advanced gastric cancer and would meet the Japanese Gastric Cancer Treatment Guidelines.

As shown in Figure 4A, intraperitoneally injected DFP‐10825 demonstrated prolonged retention in the peritoneal cavity compared with a free TS shRNA formulation (Figure 4A). Such a long retention of DFP‐10825 in the body cavity was also observed following intrathecal injection into a malignant pleural mesothelioma xenograft model.^11^ In addition, interestingly, a current study showed a selective accumulation of DFP‐10825 in peritoneally disseminated tumors (Figure 4B), although the mechanism behind such selective tumor accumulation remains unclear. Figure 4 demonstrates that the distribution of fluorescent‐labeled TS shRNA was highly consistent with that of cationic liposomes containing DiR, which indicates that TS shRNA was stably complexed with cationic liposomes even in the peritoneal cavity and was delivered by the cationic liposomes into the peritoneally disseminated tumor tissue. To cause a gene‐silencing effect, shRNA should be delivered to target cells in a complex formulation with a delivery carrier, because naked shRNA is easily degraded by the exposure of ribonuclease, and it does not easily penetrate the plasma membrane of target cells.^31^, ^32^, ^33^ Our cationic liposome, composed of DFP‐10825, could be a feasible viable system for the delivery of shRNA/siRNA to peritoneally disseminated tumors.

In the present study, the intraperitoneal injection of DFP‐10825 did not cause significant body weight changes compared with an untreated control (Figures 1B, 2C and 3B). Several RNAi drug candidates are known to induce strong systemic adverse effects as a result of inducing a cytokine storm, which is caused via stimulation of a Toll‐like receptor family that is sensed by nucleic acid molecules.^34^, ^35^ The induction of such innate immune responses is known as a bottleneck in the clinical development of RNAi drugs. Meanwhile, to avoid such an intrinsic issue, the siRNA of Patisiran (ONPATTRO^®^), the first approved therapeutic form of RNAi^36^ that was originally formulated in lipid nanoparticles,^37^, ^38^, ^39^ is directly conjugated with an *N*‐acetylgalactosamine in this formulation, which can facilitate the delivery of RNAi molecules to hepatocytes.^40^ DFP‐10825, an RNAi formulation for local administration, avoids such systemic adverse events, because very little of the TS shRNA in the DFP‐10825 formulation escapes into blood circulation from the peritoneal cavity after intraperitoneal injection (Figure 4A), and as a result, the formulation does not accumulate in immune organs such as the spleen (Figure 4B). These results indicate that DFP‐10825 for local injection is a safer RNAi formulation than other RNAi formulations.

We recently reported that DFP‐10825 also induced tumor growth suppression in a malignant pleural mesothelioma xenograft model^11^ and in a peritoneal disseminated ovarian cancer xenograft model.^30^ In the current study, DFP‐10825 also showed a superior suppressive effect on tumor growth in a peritoneally disseminated gastric cancer xenograft model (Figures 1, 2, 3). Malignant pleural mesothelioma is a cancer that develops in the pleural cavities. The life expectancy of patients with pleural mesothelioma is often less than 15 months.^41^, ^42^ Peritoneal dissemination is frequently caused by recurrent abdominal malignancy in patients with gastrointestinal cancer,^21^, ^22^ ovarian cancer,^43^, ^44^, ^45^ or pancreatic cancer,^46^, ^47^, ^48^ resulting in a poor prognosis for patients. With a great deal of continuous effort to improve the chemotherapeutic regimens, the prognosis of these advanced cancers has currently improved.^49^, ^50^ However, alternative approaches to further improve the prognosis are urgently needed. DFP‐10825 is an RNAi formulation for local administration that is capable of inducing tumor growth suppression by delivering RNAi molecules to target tumor cells such as the pleural mesothelioma cells and peritoneal disseminated cells that exist in thoracic or abdominal cavities. DFP‐10825 might be a reliable alternative to the traditional chemotherapeutic regimen via suppression of tumor growth without severe adverse effects, which should achieve a higher therapeutic index.

## 5 CONCLUSIONS

Herein, we provide the first description of intraperitoneally injected DFP‐10825, a conjugate of TS shRNA with cationic liposomes, which produced a dose‐dependent therapeutic effect on a peritoneally disseminated gastric cancer model. The growth inhibitory effect of DFP‐10825 on peritoneally disseminated tumors was superior to a conventional S‐1 treatment. Importantly, treatment with DFP‐10825 showed no systemic adverse effects. These results strongly indicate that intraperitoneal treatment with DFP‐10825 would provide a new therapeutic approach that could improve the outcomes for patients with advanced gastric cancer.

## CONFLICT OF INTEREST

MF and KE are employees of Delta‐Fly Pharma, Inc. The authors report no other conflicts of interest in this work.
